# Clinical characteristics and mortality in all Czech patients after pacemaker implantation in the last decade

**DOI:** 10.3389/fcvm.2023.1248145

**Published:** 2023-12-08

**Authors:** Miloš Táborský, Tomáš Skála, Ladislav Dušek, Josef Kautzner, Renata Aiglová, Marián Fedorco, Jiří Jarkovský, Klára Benešová, Petra Májková

**Affiliations:** ^1^ Department of Internal Medicine I—Cardiology, University Hospital Olomouc, Olomouc, Czech Republic; ^2^ Institute of Health Information and Statistics of the Czech Republic, Praha, Czech Republic; ^3^ Cardiac Centre, Institute for Clinical and Experimental Medicine—IKEM, Praha, Czech Republic; ^4^ Faculty of Medicine, Institute of Biostatistics and Analyses, Masaryk University, Kamenice, Czech Republic

**Keywords:** pacemaker, implantation, Czech republic, mortality, completeness, registry

## Abstract

**Background and aims:**

Analysis of mortality from the national health registries and data from a specific central registry dealing with the implantation of pacemakers (REPACE) in Czech patients.

**Methods and results:**

Retrospective observational analysis of pacemakers’ implantation in all Czech patients [*n *=* *82,791; 47,070 (56.9%) men, 75.9* *±* *10.4 years old] between 2010 and 2021. Almost 114,000 pacemakers were implanted between 2010 and 2021, of which 27.9% were single-chamber, 67.4% were dual-chamber and 4.6% were biventricular. The annual number of implantations has been steadily increasing with a 6% annual decline in 2020 with increased mortality and reductions in care provided, likely related to COVID-19. The observed 5-year relative survival was 88.6% (overall survival 60.6%) and the 10-year relative survival was 75.9% (overall survival 32.7%). Causes of death 5ary according to the age of the patient. The highest difference 1n the reported numbers in the REPACE Registry did not exceed 2% in comparison with the National Register of Reimbursed Health Services.

**Conclusion:**

This study followed all Czech patients with pacemaker’s implantation in between 2010 and 2021. The annual number of 1mplantations has been steadily 1ncreasing. Patients with implanted pacemakers had a significantly higher mortality than the average population. Number of patients in the registry corresponded almost perfectly with the National Register of Reimbursed Health Services.

## 1. Introduction

Effective monitoring of healthcare interventions and efficient allocation of resources can be achieved through the implementation of national registries (1). However, managing and establishing these registries can be challenging as data inadequacies and insufficiencies may arise, which require significant investments in terms of finances and human resources to ensure data accuracy and high-quality collection (2). Despite the need for mandatory participation in National Registries to obtain comprehensive data, many countries still rely on voluntary participation, which results in incomplete databases (3). The quality and completeness of such databases can also vary widely, both within and between countries (4). The Pacemaker (PM) Registry of the Czech Society of Cardiology (REPACE) collects information about demographics, clinical characteristics, main indications for PM therapy, device types, implantation details and complications from all centers in the Czech Republic. It was established in 1990 by prof. J. Lukl in Olomouc. Data entry is mandatory for implanting physicians and is a condition for payment of the procedures by the health care payers. The registry, despite being robust, does not provide data about mortality. The degree of data collection quality can be measured using the ratio between the number of procedures submitted to the registry and the number of procedures carried out within a particular geographical region, which is known as completeness (5). Despite being a frequently utilized indicator, data collection can still be inadequate even if it is mandatory (6). The National Register of Reimbursed Health Services contains all data from health insurance companies in both inpatient and outpatient areas, including complete data on reported diagnoses, procedures and treatments. When linked with the *Information System Deaths* as the primary source of information on each death, data on mortality of patients after PM implantation can be obtained.

Analysis of the completeness of the REPACE Registry and analysis of mortality of all Czech patients with a pacemaker has not yet been done.

## 2. Methodology

### 2.1. Data sources

The analysis is based on data managed by the *Institute of Health Information and Statistics of the Czech Republic* (IHIS CR), which are collected within the *National Health Information System* (NHIS) and *national health registries* combined with the data of registry of the Czech Society of Cardiology REPACE.

1. *The National Register of Reimbursed Health Services* (NRRHS) contains data from health insurance companies in both inpatient and outpatient areas, including complete data on reported diagnoses, procedures and treatments. At the time of analysis, data were available for the period 01/2010–12/2021.
2. *Information System Deaths* is the primary source of information on each death. It is completed immediately after the examination of the deceased by the examining physician, who, in addition to basic socio-demographic characteristics, also records the sequence of causes leading to death (coded using ICD-10). At the time of analysis, data were available until the end of 2021.
3. The REPACE registry aims to create a central registry for detailed clinical data of pacemaker implantation in indicated patients. At the time of analysis, data were available until the end of 2021.

### 2.2. Study population and event definition

#### 2.2.1. Identification of patients with implanted devices

In the NRRHS data, the implanted device is identified by the reported medical devices. The patient should also have a reported procedure code for the implantation performed; based on the procedure code and also on the available patient history going back to 2010, it is possible to distinguish with sufficient accuracy between 1st PM implantations and PM replacements performed.

#### 2.2.2. 1st PM implantation is defined by the procedure code

07234 (*)—Surgical implantation or replacement of permanent pacing system without epicardial leads; 17249—1st implantation of leadless pacemaker for single-chamber right ventricular pacing; 17625—1st implantation of biventricular pacing system; 17630—1st PM implantation for cardiac contractility modulation; 55211 (*)—Pacemaker implantation for single-chamber pacing; 55213—1st PM implantation for biventricular pacing. *PM replacement is defined by the procedure code:* 55219—Cardiac PM replacement without vein intervention. Some procedures (*) are non-specific and do not allow direct differentiation between 1st PM implantation and PM replacement.

## 3. Statistical analysis

Standard descriptive statistics were used for analysis. Continuous parameters were described using the mean and standard deviation, while binary or categorical parameters were described using absolute and relative numbers. Overall survival was calculated using the Kaplan-Meier method, and relative survival was calculated using the Pohar-Perme method. The probability of hospitalization for heart failure was calculated using the cumulative incidence method, with death considered as a competing event. The level of statistical significance used in all analyses was *p* = 0.05. Analyses were performed with SPSS 28.0.1.1 (IBM Corp., Armonk, NY, USA) and the R-package relsurv.

## 4. Results

### 4.1. Analysis of data from NRRHS

#### 4.1.1. Sex and age structure of patients at PM implantation (2010–2021)

A total of 82,791 patients underwent 1st PM implantation (47,070 males (56.9%); 35,721 females (43.1%)). The mean age at the time of implantation was 75.9 ± 10.4 (median 77, IQR 71–83). The mean age for males was 74.8 ± 10.3 (median 76, IQR 69–82). The mean age for females was 77.3 ± 10.2 (median 79, IQR 72–84). Generally, a higher proportion of pacemakers are implanted in men (56.9% vs. 43.1% in women). This proportion is increasing over time: in 2010, the proportion of men was 55.3%; in 2021, it is 57.8%. From 2010 to 2021, the average age of patients at implantation increased by 1 year from 75.3 years to 76.3 years.

#### 4.1.2. Selected patient comorbidities at 1st PM implantation (2010–2021; *N* = 82 791)

Diabetes mellitus (29.1%), Hypertension (84.4%), ischemic heart disease (hospitalization history/PCI/CABG) (18.7%), Heart failure (hospitalization history) (13.8%), Stroke (hospitalization history) (6.4%), Cancer (diagnosis in the last 5 years, malignant neoplasms except C44—melanoma) (5.3%).

#### 4.1.3. Number of implanted devices

**Figure 1:**
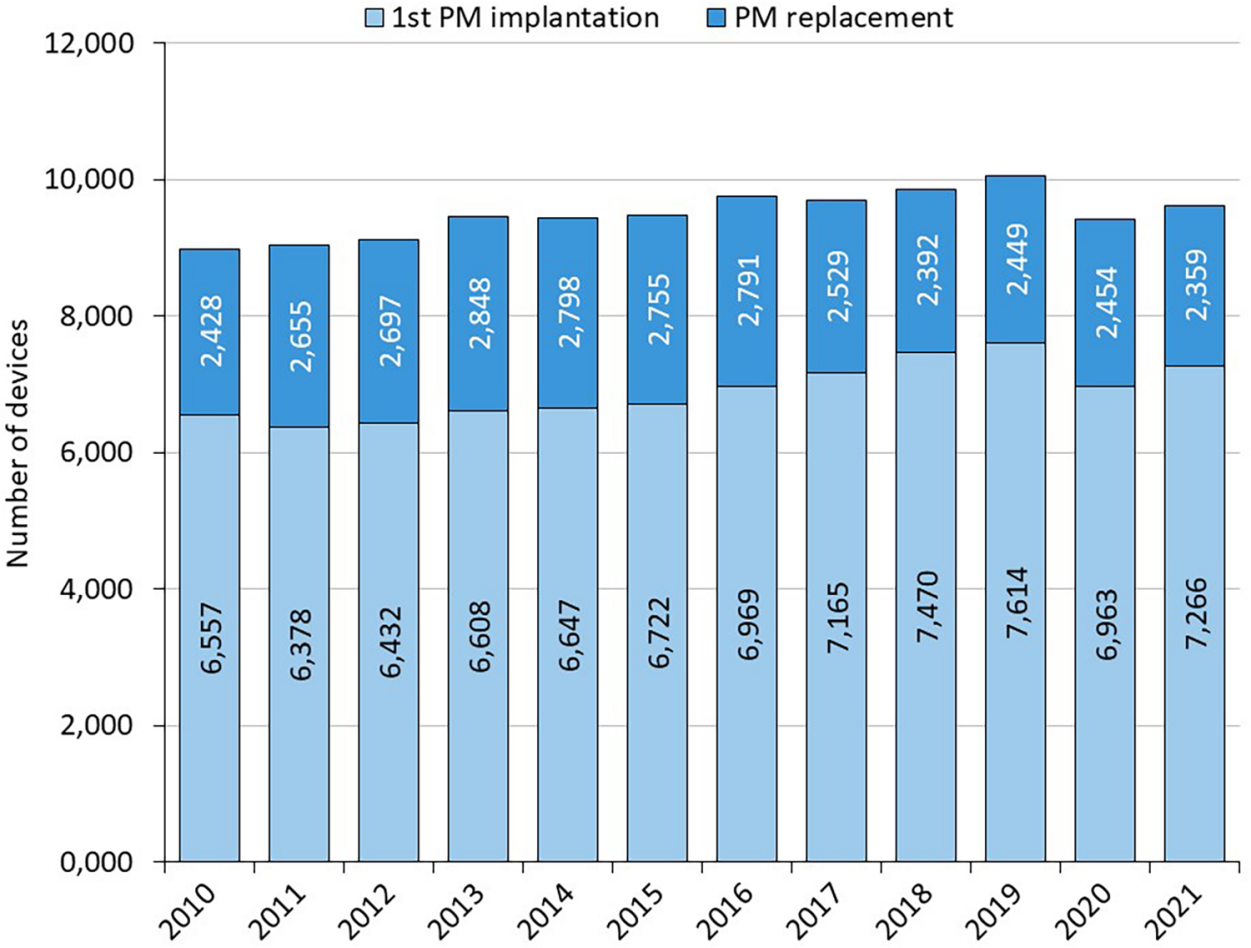
Number and type of all implanted devices 2010–2021.

**Figure 2:**
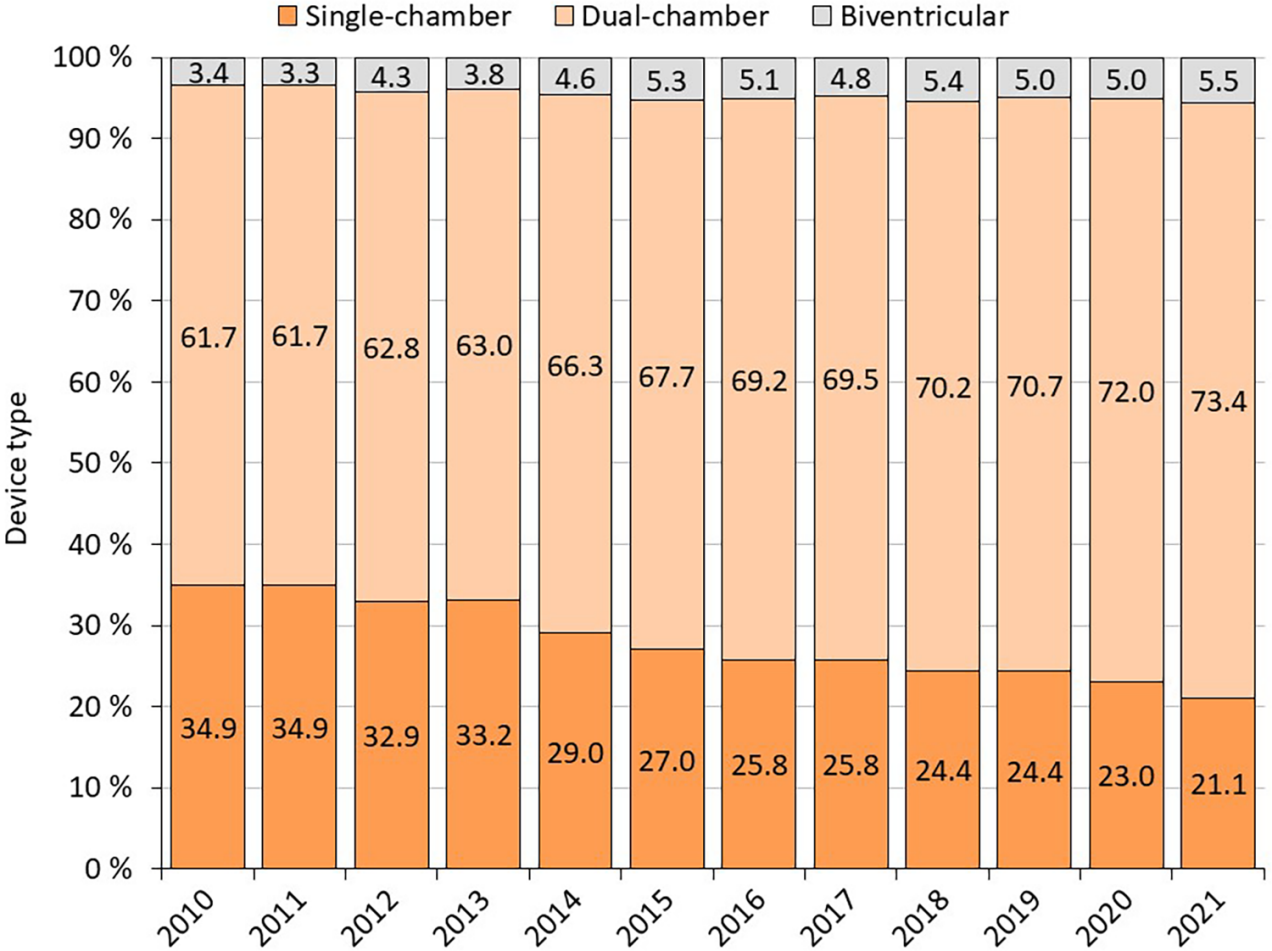
Type of implanted devices in 2010–2021.

1st PM implantation/PM replacement: the annual number of PM implantations is steadily increasing slightly (on average 120 cases per year between 2010 and 2019) (Figure 1 and Table 1). In 2020, there is a 6% annual decline in number of pacemaker implantations, likely related to COVID-19, increased mortality and reductions in care provided. The annual proportion of PM replacements to total procedures performed ranges from 24% to 30%. Almost 114,000 pacemakers were implanted between 2010 and 2021, of which 27.9% were single-chamber, 67.4% were dual-chamber and 4.6% were biventricular (Figure 2).

**Table 1:** Number of pacemaker implantation per 100,000 inhabitants and standardized to the 2013 European standard population (ESP).

Year	Absolute number of PM	Number per 100,000 citizens (ESP 2013)			
		PM—total	Single-chamber	Dual-chamber	Biventricular
2010	8.985	108.2	40.9	63.9	3.4
2011	9.033	105.2	38.9	63.1	3.3
2012	9.129	104.4	36.3	63.9	4.3
2013	9.456	105.6	36.6	65.1	3.9
2014	9.445	103.8	31.6	67.5	4.7
2015	9.477	102.2	28.9	68.0	5.3
2016	9.76	102.5	27.6	69.8	5.1
2017	9.694	99.7	26.9	68.2	4.6
2018	9.862	98.9	25.0	68.6	5.3
2019	10.063	99.2	25.2	69.1	4.9
2020	9.417	91.5	21.9	65.1	4.5
2021	9.625	92.5	20.3	67.1	5.1

#### 4.1.4. 5-year and 10-year overall survival of patients after 1st PM implantation

**Figure 3:**
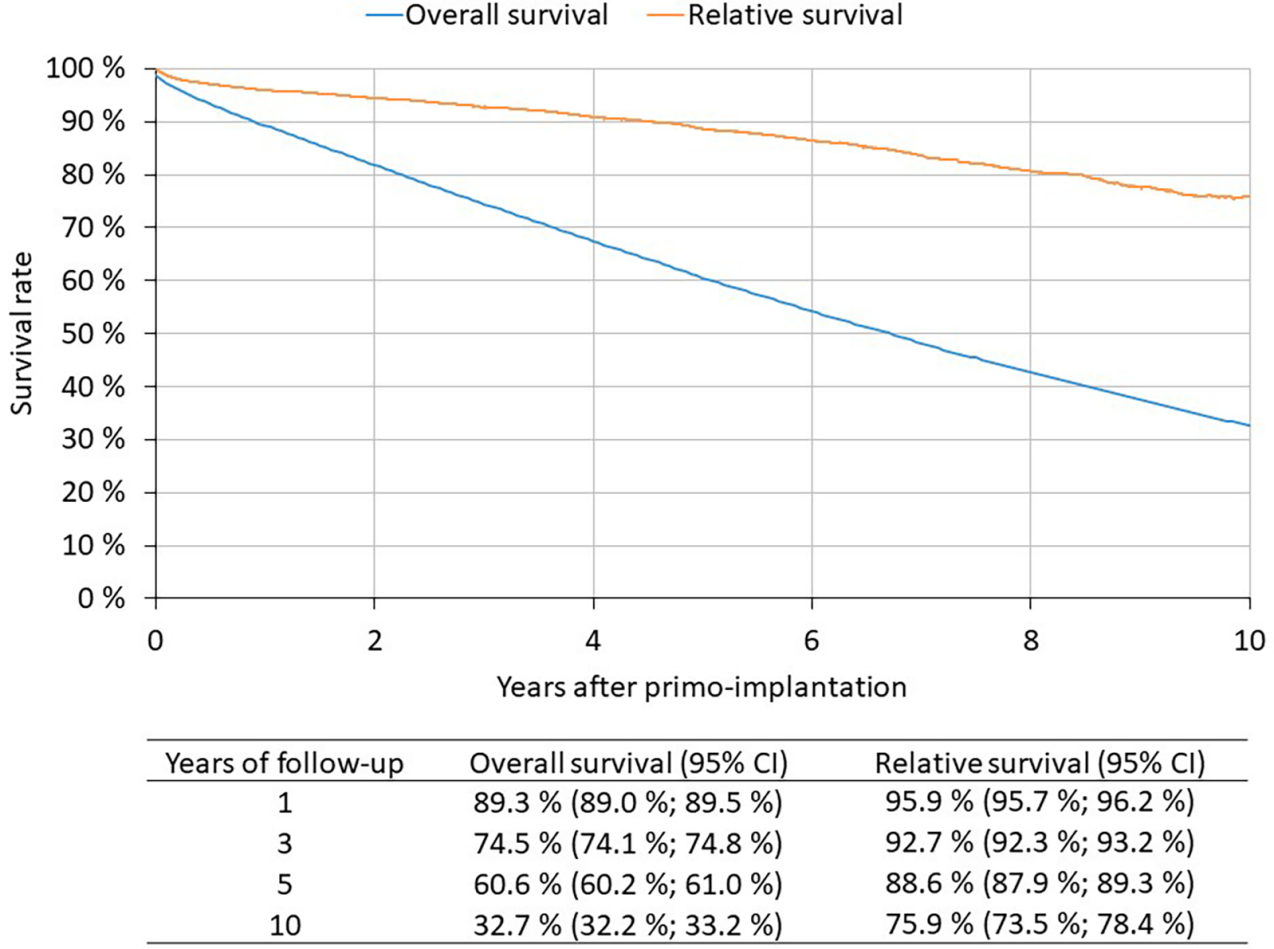
Overall and relative survival of patients after 1st PM implantation.

1st PM implantation patients 2010–2021 (*N* = 82,791): 60.6% of patients live to 5 years after 1st PM implantation, 32.7% live to 10 years (Figure 3). The Kaplan-Meier method is used to assess survival. The patient is followed from the date of 1st PM implantation to the date of death. If death is not recorded, the patient is censored on 31 December 2021.

#### 4.1.5. 5-year and 10-year relative survival of patients after 1st PM implantation

1st PM implantation patients 2010–2021 (*N* = 82,791): Relative survival reflects the survival rate of patients with implanted pacemakers compared to the expected survival of a comparable group in the general population (Figure 3). 5-year relative survival is 88.6%, 10-year relative survival is 75.9%. Relative survival was estimated by the Pohar-Perme method; observed survival was determined by the Kaplan-Meier method, expected survival was based on mortality tables for the Czech population.

Survival according to pacemaker type can be found in (Supplementary Figure S1). The worst survival rate had patients with single-chamber PM, patients with CRT-P had a slightly better survival rate; patients with dual-chamber had the best survival rate.

Hospitalizations for heart failure in patients after 1st PM implantation: The highest rate of hospitalizations for heart failure had patients with biventricular pacemakers, followed by single-chamber PM patients; the lowest rate had patients with dual-chamber pacemakers.

According to the years of follow-up after first PM implantation, the probability of hospitalization for heart failure (95% CI) was following: 1 year = 7.9% (95% CI 7.7%–8.1%); 3 years = 14.7% (95% CI 14.4–15.0%); 5 years = 19.5% (95% CI 19.2–19.8%); 10 years = 26.9% (95% CI 26.5–27.3%).

#### 4.1.6. Overall survival of patients after 1st PM implantation by age

**Figure 4:**
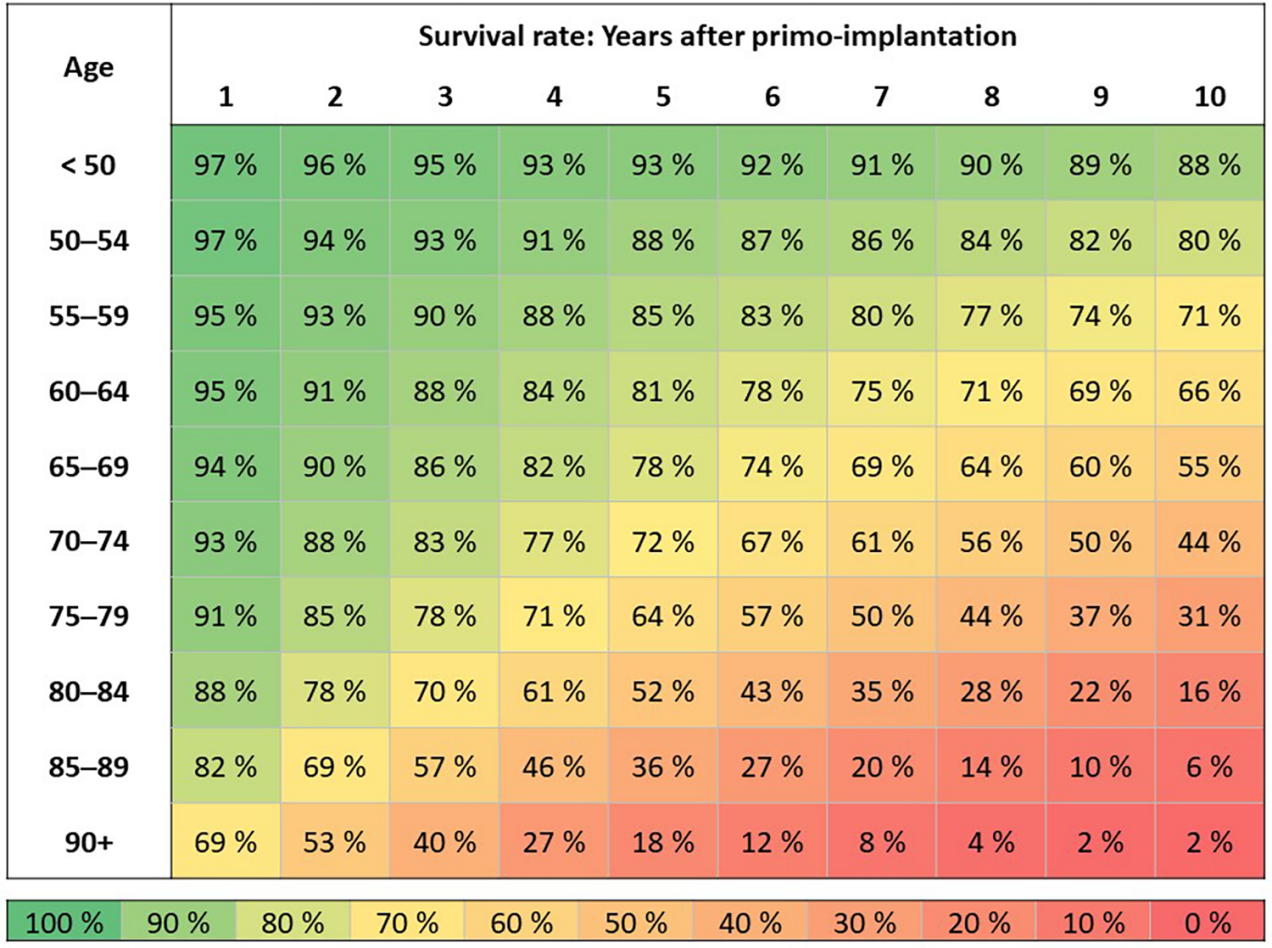
Overall survival of patients after 1st PM implantation by age.

Patients with 1st PM implantation in 2010–2021 (*N* = 82,791) according to the age of the patient at the time of surgery: Survival duration is assessed by the Kaplan-Meier method (Figure 4). Patients are followed from the date of 1st PM implantation to the date of death. If death was not recorded, the patient is censored as of 12/31/2021.

#### 4.1.7. Causes of death in persons with implanted PM

In 2010–2019, the most common cause of death in persons with implanted PM were cardiovascular diseases (62% of deaths; 31% dg. I25 chronic ischemic heart disease, 6% dg. I50 heart failure, 4% dg. I21 acute myocardial infarction). This is followed by oncological (13%) and respiratory diseases (7%). The average age of the deceased was 83 ± 8 years. In the years 2020–2021 affected by the COVID-19 pandemic, cardiovascular diseases remained the most common cause of death in persons with implanted PM (51% of deaths; 25% dg. I25 chronic ischemic heart disease, 7% dg. I50 heart failure, 2% dg. I63 cerebral infarction) followed by COVID-19 (13%) and oncological diseases (11%). The average age of the deceased was 84 ± 8 years. Causes of death vary according to the age of the patient; the proportion of deaths due to diseases of the circulatory system increases with age, whereas the proportion of deaths due to cancer decreases.

### 4.2. Analysis of data from REPACE

37 centers are performing implantations and contributing to the national Registry. The most clinically relevant parameters from REPACE Registry 2010 and 2021 are summarized in Table 2 (data from all years available in the Supplementary Table S1). In the last evaluated year (2021), there were 10,096 procedures in total [7,738 1st PM implantations (76.6%); 2,079 PM replacements (20.6); 145 device upgrades (1.4%); 28 device explantations (0.3%); 57 electrode substitutions (0.6%); 27 electrode repositions (0.3%); 14 electrode extraction (0.1%) and 8 procedures described as other surgery (0.1%)]. Of all implanted devices, 2,482 (24,9%) were single-chamber, 6,971 (70,0%) dual-chamber and 505 (5,1%) were biventricular. In Table 2, only 1st PM implantations and PM replacements are included. The other above-mentioned procedures are excluded from analysis.

**Table 2:** Characteristics of pacemaker implantations (data from the REPACE registry).

Year	2010	2021
*N*	9060	9817
Gender		
Female	4892 (54.0%)	5666 (57.7%)
Male	4168 (46.0%)	4151 (42.3%)
Age		
Mean ± SD	74.9 ± 11.3	75.9 ± 11.2
Median (5th-95th percentile)	77 (56; 88.0)	77 (57; 90.0)
Height*		
Mean ± SD	168.5 ± 10.8	169.3 ± 11.4
Median (5th-95th percentile)	169 (153; 184.0)	170 (153; 185.0)
Weight*		
Mean ± SD	79.4 ± 16.4	82.7 ± 19.3
Median (5th–95th percentile)	79 (55; 108.0)	82 (55; 115.0)
Etiology		
IHD without IM	4 177 (46.1%)	3 002 (30.6%)
IHD after IM	1 197 (13.2%)	672 (6.8%)
Aortic valve disease	303 (3.3%)	421 (4.3%)
Mitral valve disease	276 (3.0%)	227 (2.3%)
Previous cardiochirurgy	194 (2.1%)	240 (2.4%)
Dilatative cardiomyopathy	170 (1.9%)	114 (1.2%)
Previous carotid sinus syndrome	107 (1.2%)	202 (2.1%)
Other cardiomyopathy	45 (0.5%)	85 (0.9%)
Othera valve disease	48 (0.5%)	97 (1.0%)
Inborn cardiac disorder	28 (0.3%)	54 (0.6%)
Hypertrophic cardiomyopathy	30 (0.3%)	36 (0.4%)
His bundle ablation	23 (0.3%)	15 (0.2%)
Myocarditis	28 (0.3%)	11 (0.1%)
Long QT syndrome	9 (0.1%)	7 (0.1%)
ARVD/cardiomyopathy	2 (0.0%)	3 (0.0%)
Burgada syndrome	0 (0.0%)	2 (0.0%)
Short QT syndrome	0 (0.0%)	0 (0.0%)
Other etiology	1 049 (11.6%)	1 255 (12.8%)
Symptoms		
Bradycardia	3 707 (40.9%)	6 128 (62.4%)
Syncope	2 983 (32.9%)	2 308 (23.5%)
Presyncope	1 637 (18.1%)	1 139 (11.6%)
Dizziness	1 098 (12.1%)	539 (5.5%)
Congestive failure	809 (8.9%)	737 (7.5%)
Tachycardia	270 (3.0%)	540 (5.5%)
Palpitation	449 (5.0%)	290 (3.0%)
Brain dysfunction	209 (2.3%)	70 (0.7%)
Circulatory arrest	110 (1.2%)	146 (1.5%)
Prophylaxis	129 (1.4%)	17 (0.2%)
Stenocardia	55 (0.6%)	42 (0.4%)
Other symptoms	311 (3.4%)	252 (2.6%)
No symptoms	727 (8.0%)	388 (4.0%)
Heart rhythm before surgery		
Sinus	4 700 (51.9%)	4 052 (41.3%)
Bradycardia	1 554 (17.2%)	2 673 (27.2%)
Chronic fibrillation	1 770 (19.5%)	1 763 (18.0%)
Paroxysmal fibrillation	798 (8.8%)	863 (8.8%)
Unknown	46 (0.5%)	43 (0.4%)
Other rhythm	715 (7.9%)	591 (6.0%)
Vessel disease*		
No vessel disease	990 (44.2%)	1 163 (55.4%)
1VD	409 (18.3%)	315 (15.0%)
2VD	287 (12.8%)	244 (11.6%)
MVD	552 (24.7%)	378 (18.0%)
NYHA		
I	1 633 (22.2%)	1 757 (28.7%)
II	3 819 (52.0%)	3 539 (57.8%)
III	1 590 (21.6%)	788 (12.9%)
IV	303 (4.1%)	39 (0.6%)
Ejection fraction left ventricle*		
≤25%	133 (5.0%)	50 (2.0%)
26%–35%	164 (6.2%)	101 (4.1%)
36%–45%	447 (16.8%)	263 (10.6%)
46%–55%	841 (31.6%)	872 (35.1%)
56%–65%	922 (34.7%)	1 083 (43.7%)
>65%	153 (5.8%)	112 (4.5%)
Main diagnosis		
Intraventricular conduction defects	154 (1.7%)	90 (0.9%)
Cardiomyopathy	175 (1.9%)	135 (1.4%)
Conduction disorder	3 345 (36.9%)	4 852 (49.4%)
Valve disease	212 (2.3%)	223 (2.3%)
Sick sinus syndrome	3 338 (36.8%)	2 929 (29.8%)
Vasovagal syncope	46 (0.5%)	40 (0.4%)
Hypersensitive carotid sinus	84 (0.9%)	29 (0.3%)
IHD—atrial bradycardia	1 304 (14.4%)	1 102 (11.2%)
IHD—ventricular tachycardia	22 (0.2%)	11 (0.1%)
IHD—conduction disorder, SSS	355 (3.9%)	159 (1.6%)
Unknown	25 (0.3%)	247 (2.5%)
Device type 1		
1D	3 295 (36.4%)	2 471 (25.2%)
2D	5 422 (59.9%)	6 946 (70.8%)
BiV	253 (2.8%)	386 (3.9%)
VDD	87 (1.0%)	1 (0.0%)
Device type 2		
Leadless	0 (0.0%)	66 (0.7%)
Other	9 060 (100.0%)	9 751 (99.3%)

**Figure 5:**
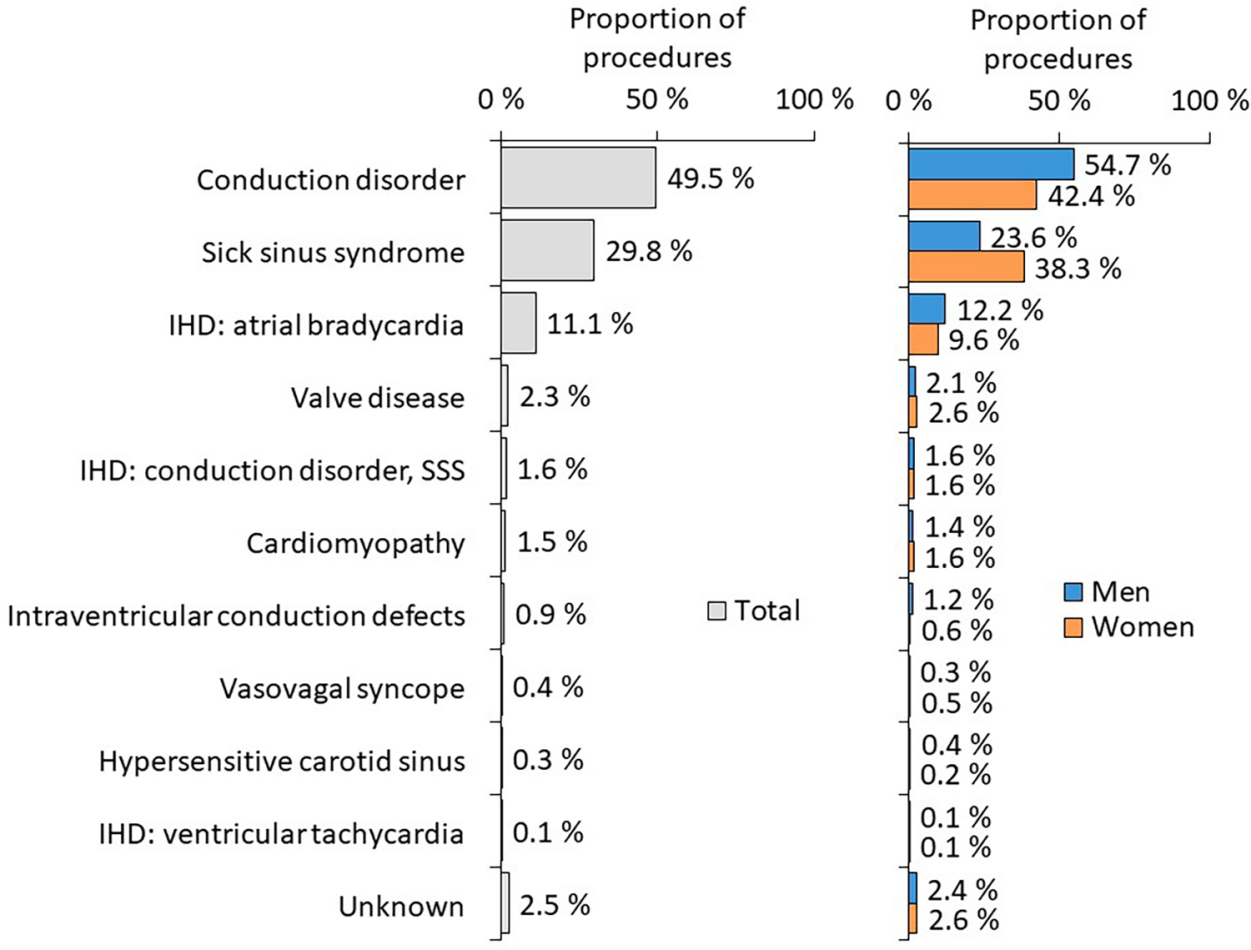
The most frequent diagnoses at the time of PM implantation according to sex.

The most frequent diagnoses at the time of PM implantation according to sex are summarized in Figure 5.

*Surgery complications* were quite rare. There were almost no complications during 1st PM implantation or PM replacement. In 2021, during 1st PM implantation or PM replacement, respectively, the implanting physicians reported electrode displacement in 0,3% and 0,2%, pneumothorax in 0,1% and 0,1%, technical complication in 0,1% and 0%, pocket hematoma in 0,1% and 0,1%, electrode penetration / perforation in 0,1% and <0,1%, hemothorax in <0,1% and 0%, early infection in <0,1% and 0,1%, electrode displacement in 0% and 0%.

#### 4.2.1. Reported number of implantations in national register of covered health services and REPACE comparison

When comparing the number of devices (1st PM implantations and PM replacements) in the National Register of Reimbursed Health Services and the number reported to the REPACE Registry, the numbers did not exceed 2% in any year from 2010 to 2021.

## 5. Discussion

Registries are increasingly becoming mandatory, but implanting physicians may find data entry into such registries burdensome, especially given the growing bureaucracy in healthcare. Moreover, they may be apprehensive about declaring their procedural volume and complications. Despite this, current quality standards necessitate such documentation. To ensure compliance, it is essential that registries are designed in a user-friendly manner that streamlines data entry and offers adequate incentives to users. Encouraging users to input complete and accurate data is a significant obstacle for national registries, as such data is fundamental for meaningful epidemiological analysis and clinical research.

In the Czech Republic, The REPACE Registry provides a full overview of the recorded implantable devices, their attributes, and some information relating to patients’ personal and clinical data. Since data are collected on a compulsory basis, the Registry completeness is high. This is reflected in a very similar number of reported devices implantation in data from the *National Register of Reimbursed Health Services* and the *REPACE* Registry. The highest difference in the reported numbers in the *REPACE* Registry did not exceed 2% in comparison with the *National Register of Reimbursed Health Services.* Although the current study is a retrospective analysis derived from a registry, which has its limitations, the data collection was conducted prospectively by the implanting physicians and is therefore expected to be of superior quality.

The vast majority of clinically relevant data can be derived from the REPACE registry. In the Czech patients, the annual number of PM implantations has been steadily increasing slightly (on average 120 cases per year between 2010 and 2019). The gradual increase in the number of patients can be explained by the ageing of the population (7). From 2010 to 2021, the average age of patients at implantation increased by 1 year from 75.3 years to 76.3 years. The gradual ageing of the population and the corresponding increase in the number of PMs is also evident in other countries (8). According to the results of the 2021 Census, the population of the Czech Republic was 10,524,167. In 2021, 9,625 PMs were implanted in the Czech Republic, which corresponds to 91.46 PMs per 100,000 inhabitants. Total implantation numbers per 100,000 inhabitants vary in Europe widely, from Germany (196.53 per 100.000 inhabitants) to Kosovo (2.81 per 100.000 inhabitants). Higher implantation numbers correlate moderately with a higher GDP and higher health expenditure. The Czech Republic is somewhere in the middle and is similar to Spain (95.3 per 100.000 inhabitants) and Luxembourg (91.7 per 100.000 inhabitants) (9).

After standardizing our data to European Standard Population (ESP) that includes balancing the age structure so that values across countries and between years are comparable, we have observed that as the country’s population ages and the absolute number of implantations per year increases only slightly, the number of implantations converted to 2013 ESP is decreasing.

A higher proportion of pacemakers were implanted in men. This proportion has been increasing over time. The proportion of men in this population is very similar to other registries (8, 10).

Of all implanted devices, 27.9% were single-chamber, 67.4% were dual-chamber and 4.6% were biventricular (11). The ration of biventricular PMs (CRT-P) is higher than in Netherlands [450 CRT-Ps of 10,000 PMs in 2016 (4,5%)], Spain [1124 CRT-Ps of 37,466 PMs in 2016 (3,0%)] and even Germany (3700 CRT-Ps of 110 100 PMs in 2016 (3,4%) (12). The ratio of 1st PM implantation to PM replacements rose steadily during the whole decade. One reason for this might be improvements in PM battery longevity, confirming data observed in the AIAC Registry (13). When comparing our results from the HDR analysis with the AIAC Registry data for 2017, similar proportions of patients with AV block and sick sinus syndrome were observed (13). The occurrence of ischemic heart disease was noticed to be higher in 1stPMs compared to other registries. The difference can be attributed to the fact that the European PM card permits only one diagnosis/etiology to be recorded, whereas in in the REPACE Registry, several different diagnoses, not necessarily linked to the procedure, were accepted. It is possible that the other registries have underestimated the incidence due to this limitation (8, 13).

Surgery complications were quite rare (<1%) as there were almost no complications during 1st PM implantation or PM replacement. This is a far better result than previously reported numbers of complications in Medicare beneficiaries, where complications during the index hospitalization occurred in 7,046 patients (5.18%), and complications within 90 days of device implantation occurred in 10,005 patients (7.34%) (14).

Despite providing detailed data, the REPACE registry is not linked with data on mortality. These can be derived from the *National Register of Reimbursed Health Services* since this registry can be linked with *Information System Deaths, contrary to the Repace registry.* In 2020, there was a 6% annual decline in number of pacemaker implantations, likely related to COVID-19, increased mortality and reductions in care provided. More than 60% of patients live to five years after 1st PM implantation, and more then 32% are alive after ten years. The observed 5-year relative survival was 88.6% and the 10-year relative survival was 75.9%. The most frequent cause of death in Czech patients with implanted PM are cardiovascular diseases, followed by cancer and respiratory diseases. The average age of the deceased was 83 years. This is in correspondence with the fact that the average age of Czech patients at the time of pacemaker implantation is 76 years. In the last decade, the average age of patients at implantation increased by 1 year. In the years 2020–2021 affected by the COVID-19 pandemic, cardiovascular diseases remained the most common cause of death in persons with implanted PM. However, they were followed by COVID-19 as the second most common cause of death. Causes of death varied according to the age of death of the patient. It is well known, that the survival of patients with pacemakers is independently influenced by several baseline characteristics which can identify patients with very long survival (15). The proportion of deaths due to circulatory diseases increased with age, whereas the proportion of deaths due to cancer decreased.

The worst survival rate had patients with single-chamber PM, patients with CRT-P had a slightly better survival rate; patients with dual-chamber had the best survival rate. Explanation of this is probably in the fact that patients with a single-chamber PM were 6 years older in average. Age structure of dual-chamber and CRT-P patients was similar, however patients with biventricular pacemakers tend to have more severe health issues so their higher mortality could have been expected.

The highest rate of hospitalizations for heart failure had patients with biventricular pacemakers, followed by a significantly older subgroup of single-chamber PM patients. The lowest rate of heart failure hospitalizations had patients with dual-chamber pacemakers. Within 5 years of first PM implantation, every 5th patient was hospitalized at least once for heart failure.

Given the lack of integration of existing clinical data for patients with implanted PM with mortality data, a major modification of the REPACE registry, with automatic integration of data from the *Information System Deaths*, is necessary in the future.

Two separate registries that could not have been directly linked and analyzed together were administered in the beginning of data collection on device implantations in the beginning and thus the REPACE registry does not contain data on ICD implantations. To correct this fact an integration of the PM and ICD registries will be performed in the very near future. The registries also contain a lot of information that is already obsolete, and conversely, many important parameters are not available. The following basic information will be mandatory for each patient: patient identification, hospital, procedure, preoperative information including clinical characteristics and symptoms, EHRA diagnosis, associated diseases and previous procedures, heart rhythm, ECG diagnosis and LV examination. Operative information will include basic information about the procedure, generator implantation/explantation, electrode implantation/explantation, and postoperative status information, including mortality data.

Currently, the reporting of complications associated with PM implantation is <0.9% of all procedures in the long term and is undoubtedly underreported. In the new structure of the register, from 2023 on, significantly more attention is paid to this issue.

### 5.1. Study limitations

Patients in the National Register of Reimbursed Health Services and the REPACE registry are not directly linked. On the other hand, the total numbers of PM implantations are almost identical, indicating excellent physician compliance in completing the REPACE registry. As stated above, the number of patients in the registry corresponded almost perfectly with the data of National Register of Reimbursed Health Services.

It is possible that certain codes may be open to interpretation, such as PMs code 07234 and 55211 which are non-specific and do not allow direct differentiation between 1st PM implantation and PM replacement.

Only single-chamber, dual-chamber a biventricular devices discrimination is available and no in-depth analysis of AAI vs. VVI and VVI vs. DDD and/or CRT-P pacemakers is obtainable. Moreover, with regard to the analyzed timeline, the cardiac pacing register does not analyze the issue of pacing in the area of the cardiac conduction system, which has been gradually developing in the Czech Republic for the last five years (16). However, there was no observable decrease in CRT-P implantations number until 2021 that could have been linked to an increase in conduction system pacing. At this time, we consider it very important that the EHRA consensus on this issue has been published and thus creates a clear direction for the future in this certainly very promising area of cardiac pacing (17).

## 6. Conclusion

This study followed all Czech patients with pacemaker’s implantation in between 2010 and 2021. The annual number of implantations has been steadily increasing. Patients with pacemakers had a significantly higher mortality than the average population. Number of patients in the registry corresponded almost perfectly with the data of National Register of Reimbursed Health Services.

## Data Availability

The original contributions presented in the study are included in the article/**Supplementary Material**, further inquiries can be directed to the corresponding author.

## Supplementary material

SUPPLEMENTARY TABLE S1

Characteristics of pacemaker implantations (data from the REPACE Registry).

SUPPLEMENTARY FIGURE S1

Overall survival of patients after 1st PM implantation according to the pacemaker type.

SUPPLEMENTARY FIGURE S2

Cumulative incidence of hospitalizations for heart failure in patients after 1st PM implantation according to the pacemaker type.
