# Supplementary figures and images for: Clinical characteristics and mortality in all Czech patients after pacemaker implantation in the last decade
Source: Front Cardiovasc Med. 2023 Dec 8;10:1248145. doi: 10.3389/fcvm.2023.1248145 (PMC10739293; doi:10.3389/fcvm.2023.1248145)

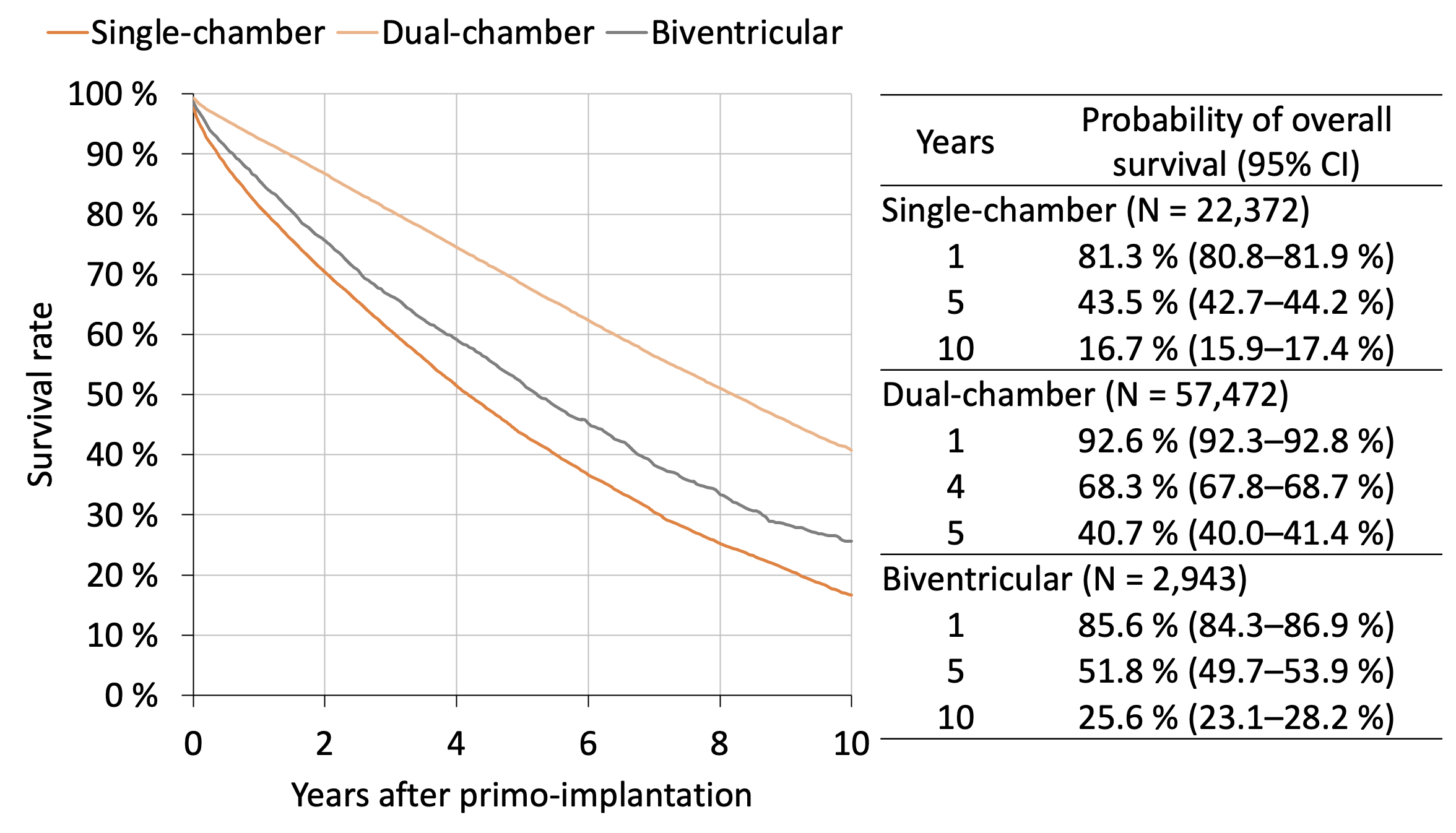

Supplement: Supplementary file 1 [file Image1.png]

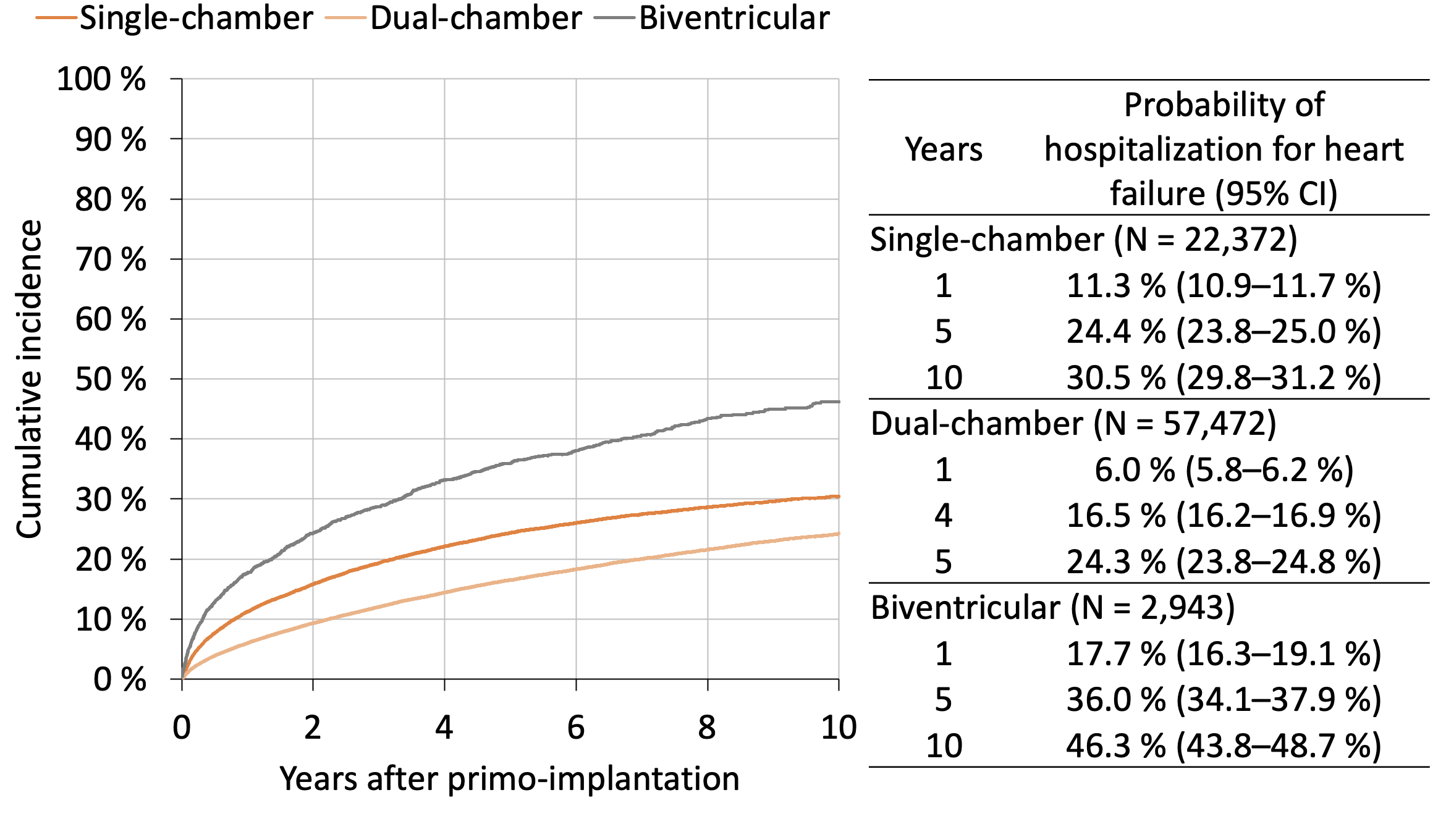

Supplement: Supplementary file 2 [file Image2.png]
